# Low-Dimensional Nano-Scaled Materials: From Principles to Device Application

**DOI:** 10.3390/mi17101138

**Published:** 2026-09-30

**Authors:** Sake Wang, Nguyen Tuan Hung

**Affiliations:** 1 College of Science, Jinling Institute of Technology, 99 Hongjing Avenue, Nanjing 211169, China; 2 Frontier Research Institute for Interdisciplinary Sciences, Tohoku University, Sendai 980-8578, Japan; nguyen.tuan.hung.e4@tohoku.ac.jp

## 1. Introduction

Low-dimensional and nano-scaled materials have attracted sustained interest because their reduced dimensionality, large surface-to-volume ratios, tunable electronic structures, and distinctive mechanical, optical, and chemical properties provide new opportunities for microsystems and nanosystems [1]. These characteristics have enabled advances in sensing, electronics, optoelectronics, energy conversion and storage, and biomedical technologies [2,3]. Nevertheless, translating nanoscale properties into practical applications requires coordinated optimization of material composition, morphology, interfaces, processing conditions, and device architecture [4,5,6,7,8,9,10,11,12]. The Special Issue “Low-Dimensional Nano-Scaled Materials: From Principles to Device Application” brings together ten contributions that illustrate this progression from fundamental materials principles to device-oriented applications.

The contributions cover graphene, MoS_2_, carbon nanomaterials, metal-oxide nanostructures [13], QDs [14], phase-engineered materials, and boron-containing nanocarriers. Their applications range from MEMS acceleration and acoustic sensing to plasma processing, photodetection, chemical sensing, photovoltaics, energy storage, and biomedical delivery. The contributing authors represent research communities from China, the Republic of Korea, Lithuania, and the USA. To present the collection in a scientifically coherent manner, the contributions are discussed below according to five major research directions: graphene-enabled mechanical and acoustic sensing; processing and integration of 2D materials; nano-enabled chemical and environmental sensing; low-dimensional materials for energy conversion and storage; and nanomaterials for precision biomedical applications [15,16,17].

## 2. Graphene-Enabled Mechanical and Acoustic Sensing

Graphene is particularly attractive for MEMS sensing because its electrical response can be strongly coupled to mechanically induced strain [18,19,20,21,22,23]. Wei et al. [24] develop a graphene-based piezoresistive MEMS accelerometer for high-g applications and progressively optimize the mechanical structure by introducing a central mass, stress-concentration grooves, and symmetric masses. The optimized architecture substantially enhances the beam-root strain while maintaining a first-order natural frequency above 200 kHz and reducing cross-axis sensitivity, and the simulated strain is further connected to resistance variation and bridge output through a graphene-based piezoresistive model. The work demonstrates how structural optimization can amplify the practical sensing capability of a low-dimensional material in extreme mechanical environments.

A complementary study by Wei et al. [25] applies graphene-based piezoresistive transduction to a membrane-supported beam–island diaphragm for high-SPL acoustic sensing. The optimized design increases the local peak strain from 47.83 με to 562.53 με at 170 dB SPL, while maintaining a highly linear pressure response, a first natural frequency of 64.07 kHz, and a small response variation over the 0–20 kHz range. Electromechanical output estimation, noise considerations, and geometric tolerance analysis further connect the structural design to practical microphone performance, highlighting the importance of integrating material sensitivity with device-level mechanical engineering.

## 3. Processing and Integration of 2D Materials for Electronics and Optoelectronics

The practical implementation of atomically thin materials depends strongly on their processing environment because plasma exposure, thermal treatment, and transfer processes can substantially influence defects and electronic properties [26,27,28]. Kim et al. [29] use 2D particle-in-cell Monte Carlo collision simulations to investigate Ar/O_2_ capacitively coupled plasmas for low-damage MoS_2_ patterning, revealing how oxygen concentration changes plasma electronegativity, ion composition, ion energy and angular distributions, and the balance between damaging ion flux and reactive oxygen species. Their analysis identifies an intermediate oxygen-fraction regime as a potentially favorable processing window, providing a mechanistic basis for controlling plasma-induced damage during fabrication.

Kumža et al. [30] investigate low-temperature direct synthesis of graphene on Si(100) at 500 °C by microwave PECVD with increased CH_4_/H_2_ flow ratio. Raman spectroscopy and atomic force microscopy reveal strong relationships among growth conditions, defects, layer number, hydrogenation, morphology, self-doping, and local conductivity, while the electrical and photoelectrical properties of graphene/Si junctions are correlated with these structural characteristics. The competitive photoresponse obtained at the reduced growth temperature demonstrates the potential of low-temperature graphene synthesis for integration with semiconductor and optoelectronic technologies.

Lee et al. [31] address device integration by developing an ion-gel-assisted transfer strategy for large-area MoS_2_ and applying it to MoS_2_/ITZO heterojunction phototransistors. The ion gel provides a high-capacitance gate dielectric, while the MoS_2_/ITZO architecture enables low-voltage phototransistor operation with a field-effect mobility of 4.12 cm^2^ V^−1^ s^−1^, an on/off ratio of $4.9\times 10^{5}$, and responsivities reaching 5.56 A W^−1^ at 405 nm. The study demonstrates how transfer, dielectric, and heterojunction engineering can translate the intrinsic optoelectronic properties of MoS_2_ into a practical device platform [32,33,34,35].

## 4. Nano-Enabled Chemical and Environmental Sensing

The large active surface area and tunable charge-transfer characteristics of nanostructured materials make them highly suitable for chemical and environmental sensing [36]. Weng and Wu [37] develop a ternary carbon composite consisting of MWCNTs, graphene, and Vulcan XC72 carbon black for simultaneous electrochemical detection of AA, DA, and UA. The composite increases the electroactive surface area and introduces defect-related catalytic sites, enabling simultaneous detection with satisfactory sensitivity, selectivity, reproducibility, and recovery in human urine samples. This work illustrates the potential of combining complementary carbon nanostructures to create synergistic electrochemical interfaces [38] for multiplexed sensing.

Yang et al. [39] develop Ce/ZnO-incorporated In_2_O_3_ nanocubes for ethanol gas sensing, using heterojunction formation and Ce-induced lattice distortion to tailor surface chemistry and charge transfer. The optimized sensor exhibits a response of 33.2 toward 100 ppm ethanol, an 8.7-fold improvement over pristine In_2_O_3_, a detection limit of 0.8 ppm, and substantially improved humidity tolerance. The combination of heterostructure engineering, lattice modification, and surface reaction control demonstrates a promising strategy for reliable gas sensing under realistic environmental conditions.

## 5. Low-Dimensional Nanomaterials for Energy Conversion and Storage

Low-dimensional materials also provide important opportunities for energy technologies because their electronic structure, interfaces, crystal phases, and morphologies can be tuned to control charge and ion transport [40,41,42]. Neupane et al. [43] review the development of QD solar cells, covering QD materials, device architectures, electron- and hole-transport layers, and recent developments ranging from lead- and cadmium-based systems to carbon- and perovskite-based QDs. The review emphasizes that further progress depends on coordinated optimization of the active layer, material interfaces, and device architecture, underscoring the importance of integrating nanoscale electronic properties with complete photovoltaic structures.

Mangiri et al. [44] review phase engineering of nanomaterials for batteries and supercapacitors, emphasizing the relationships among crystal phase, morphology, surface properties, charge transport, and ion diffusion. Their discussion includes atypical and metastable phases, mixed-phase structures, and transition-metal dichalcogenide systems, while a representative ZnCo_2_O_4_@Ti_3_C_2_ composite demonstrates a high specific capacitance, substantial reversible capacity, and more than 85% retention after 10,000 cycles. The contribution highlights phase and morphological control as powerful design parameters for improving both electrochemical performance and durability in next-generation energy-storage systems [45].

## 6. Nanomaterials for Precision Biomedical Applications

Nanoscale engineering can also address challenges that extend beyond conventional electronic and energy devices [46]. Yadav et al. [47] review nanocarrier strategies for boron delivery in BNCT, in which the short-range high-linear-energy-transfer products generated by the ^10^B neutron-capture reaction can provide highly localized tumor-cell damage. The review focuses on the limitations of conventional boron agents and discusses how nanocarriers can improve boron loading, circulation, tumor accumulation, cellular uptake, and targeted delivery. This work demonstrates how nanoscale control of material composition, surface chemistry, and transport behavior can support the development of more precise therapeutic systems.

The principal materials, device functions, representative performance metrics [48], and key materials or structural engineering strategies of the ten contributions are summarized in Table 1.

## 7. Concluding Remarks

The ten contributions demonstrate the broad scope of low-dimensional and nano-scaled materials, while also revealing several common principles that connect apparently different applications [49,50]. Graphene-based MEMS sensing relies on the coupling of nanoscale piezoresistive properties with carefully engineered mechanical architectures, whereas 2D-material optoelectronics depends on controlled synthesis, low-damage processing, transfer, and interface engineering [51,52,53,54]. Chemical and environmental sensing benefit from synergistic nanocomposites, heterojunctions, and tailored surface chemistry, while energy technologies rely increasingly on quantum confinement, interfacial control, crystal-phase engineering, and morphology optimization. The biomedical contribution further demonstrates that these nanoscale design principles can be extended to the controlled delivery of therapeutic agents [55].

Overall, this Special Issue illustrates a clear progression from materials design and processing to structure, device integration, and application. Future advances will likely depend on stronger integration of simulation and experiment [56,57,58], more precise control of interfaces [38] and defects [59], scalable fabrication methods, multifunctional architectures, and greater attention to reproducibility, stability, sustainability [60,61], and real-world operating conditions [62,63,64,65].

We sincerely thank all authors for their valuable contributions and the reviewers for their careful and constructive assessments. This edition will stimulate continued interdisciplinary collaboration, inspiring both curiosity and application-driven exploration in materials science, which we sincerely hope will contribute to the next edition of the Special Issue, “Low-Dimensional Nano-Scaled Materials: From Principles to Device Application, 2nd Edition” [66]. 

## Figures and Tables

**Table 1:** Summary of the principal materials, device functions, representative performance metrics, and key materials or structural engineering strategies reported in the contributions to the Special Issue.

Contribution	Material/System	Main Device Function	Representative Performance	Key Enabling Strategy
Wei et al. [24]	Graphene-based piezoresistor/MEMS	High-g acceleration sensing	First-order natural frequency > 200 kHz; >15× strain enhancement after mass integration	Progressive structural and strain-concentration optimization
Wei et al. [25]	Graphene-based piezoresistive MEMS	High-SPL acoustic sensing	11.8× local-strain enhancement; 64.07 kHz first resonance	Beam–island diaphragm and strain localization
Kim et al. [29]	Ar/O_2_ plasma + MoS_2_	Low-damage material processing	Identification of an intermediate-O_2_ processing window	Control of ion/radical flux balance and ion energy/angular distributions
Kumža et al. [30]	Hydrogenated graphene/Si	Photoelectric detection	Competitive photocurrent and photovoltaic parameters at 500 °C growth	Low-temperature PECVD and growth-condition control
Lee et al. [31]	MoS_2_/ITZO/ion gel	Phototransistor	Up to 5.56 A W^−1^ responsivity and $5.68\times 10^{10}$ Jones detectivity	Ion-gel-assisted transfer and heterojunction integration
Weng and Wu [37]	MWCNT/graphene/carbon black	Multiplexed electrochemical sensing	Limits of detection of 34.1, 4.23, and 11.1 μM for AA, DA, and UA	Synergistic ternary carbon composite
Yang et al. [39]	Ce/ZnO–In_2_O_3_	Ethanol gas sensing	Response of 33.2; 0.8 ppm detection limit	Heterojunction and lattice-distortion engineering
Neupane et al. [43]	QDs	Solar cells	Review of QD photovoltaic architectures and materials	Active-layer, interface, and architecture engineering
Mangiri et al. [44]	Phase-engineered nanomaterials	Batteries/supercapacitors	1013.5 F g^−1^ capacitance; >85% retention after 10,000 cycles for representative composite	Crystal-phase, morphology, and heterophase control
Yadav et al. [47]	Nanocarriers for ^10^B	Drug delivery for BNCT	Enhanced boron loading and tumor-targeting potential	Nanocarrier-mediated delivery

## Abbreviations

The following abbreviations are used in this manuscript:

2D	two-dimensional
AA	ascorbic acid
BNCT	boron neutron capture therapy
DA	dopamine
ITZO	indium–tin–zinc oxide
MEMS	microelectromechanical systems
MWCNT	multi-walled carbon nanotube
PECVD	plasma-enhanced chemical vapor deposition
QD	quantum dot
SPL	sound pressure level
UA	uric acid
